# Supportive care needs in Australian melanoma patients and caregivers: results from a quantitative cross-sectional survey

**DOI:** 10.1007/s11136-023-03492-0

**Published:** 2023-07-31

**Authors:** Jake R. Thompson, Hong Fu, Robyn P. M. Saw, Kerry A. Sherman, Victoria Beedle, Victoria Atkinson, Frances Boyle, Niamh A. O’Sullivan, Linda K. Martin, Iris Bartula

**Affiliations:** 1 Faculty of Medicine and Health, The University of Sydney, Sydney, NSW Australia; 2 Melanoma Institute Australia, The University of Sydney, 40 Rocklands Road, Wollstonecraft, Sydney, NSW 2065 Australia; 3 Department of Cancer Services, Princess Alexandra Hospital, University of Queensland, Woolloongabba, QLD Australia; 4 Department of Melanoma and Surgical Oncology, Royal Prince Alfred Hospital, Camperdown, NSW Australia; 5 Sydney Medical School, The University of Sydney, Sydney, NSW Australia; 6 School of Psychological Sciences, Macquarie University, Macquarie Park, NSW Australia; 7 Melanoma Patients Australia, Varsity Lakes, QLD Australia; 8 Patricia Ritchie Centre for Cancer Care and Research, Mater Hospital, North Sydney, NSW Australia; 9 Faculty of Medicine and Health, University of New South Wales, Sydney, NSW Australia

**Keywords:** Melanoma, Supportive care, Psycho-oncology, Quality of life, Anxiety, Unmet needs

## Abstract

**Purpose:**

This study aimed to investigate the supportive care needs of Australian melanoma patients and their caregivers to form the basis for improving services.

**Methods:**

General and melanoma-related supportive care needs in melanoma patients were measured using the SCNS-SF34 and SCNS-M12 respectively, whereas caregivers completed the SCNS-P&C. Patients also completed the MCQ-28 and FCRI-9, with all participants completing the QLQ-C30, DASS-21, and questions measuring utilisation and preference for supportive health services. Multivariable stepwise logistic regression was used to identify variables associated with unmet needs in melanoma patients.

**Results:**

A total of 56 early-stage patients, 100 advanced-stage patients, and 37 caregivers participated. At least three-quarters ($\ge$ 75%) of each participant group reported at least one unmet need. Of the ten most reported unmet needs in each participant group, at least six ($\ge$ 60%) were related to psychological and emotional well-being, with access to a psychologist the most desired service (> 25%). Fear of cancer recurrence was equally prevalent in both patient groups at a level indicative of need for intervention. Advanced-stage patients reported significantly (p < 0.05) more unmet psychological, physical and daily living, and sexuality needs, and significantly (p < 0.05) worse functioning than early-stage patients.

**Conclusion:**

Australian melanoma patients and caregivers report substantial unmet supportive care needs, particularly regarding their psychological and emotional well-being. Psychological and emotional well-being services, such as access to a clinical psychologist or implementation of patient-reported outcome measures, should be incorporated into routine melanoma care to address unmet patient and caregiver needs and improve well-being.

**Supplementary Information:**

The online version contains supplementary material available at 10.1007/s11136-023-03492-0.

## Plain English summary

As the number of melanoma survivors is increasing, there is an increased focus on the well-being and quality of life of melanoma patients and their caregivers. However, to further improve their well-being and quality of life, the unmet needs of melanoma patients and their caregivers must be identified. This study surveyed 156 melanoma patients and 37 caregivers to identify their unmet needs and assist in guiding future services to address these needs. The most common unmet needs reported by melanoma patients and caregivers related to their psychological and emotional well-being, with the most requested support service being access to a psychologist. We identified a number of melanoma patient and caregiver unmet needs, factors associated with these unmet needs, and identified differences in unmet needs and well-being between patients diagnosed with early-stage and advanced-stage melanoma. These results can be used to plan future support services to address the unmet needs of melanoma patients and caregivers in Australia.

## Introduction

Melanoma is the deadliest form of skin cancer [1]. In 2020 alone, over 300,000 individuals were diagnosed with melanoma, representing 1.7% of global cancer diagnoses [2]. The global incidence rate of melanoma has steadily increased in the past several decades, particularly in developed regions with a high proportion of fair-skinned people such as Australia, New Zealand, Scandinavia, the United Kingdom, and the United States [1]. Australia has the highest age-standardised incidence rate of 36.6 per 100,000, a rate over ten times the global average, which has earned Australia the title of ‘melanoma capital of the world’ [1, 2]*.*

Despite this, Australia reports one of the lowest melanoma mortality-to-incidence ratios of 9% [3], which can be attributed to several factors, including: (1) national campaigns to increase public awareness of sun safe and melanoma-preventative behaviours [4]; (2) improvements in the screening, imaging, surveillance, and early detection of melanoma and the implementation of high-risk clinics [5]; and (3) the advent of new and effective treatments such as immune and targeted therapies for advanced and metastatic melanoma [5]. It is anticipated that due to these ongoing advances, the number of people living with melanoma will double throughout the 2021–2030 period [5]. As such, there has been an increased focus on the supportive care of this growing cohort, particularly regarding their quality of life (QoL).

In 2021, a national survey of 1137 Australians with a previous melanoma diagnosis was conducted [5]*.* This survey found that people diagnosed with melanoma identified supportive care as one of five priority areas that should be the focus of future research and clinical care [5]. Furthermore, over 40% of respondents reported that the topic of supportive care was never raised by their treatment team, and over 50% reported that they did not receive support following the completion of treatment [5]. Thus, although substantial advances have been made in the awareness, detection, and treatment of melanoma, there is a critical need for the consideration of the supportive care needs experienced by people diagnosed with melanoma. While this report focused on the experiences of people diagnosed with melanoma, the needs of caregivers also need to be considered, as the act of caring for an individual with cancer can impact on physical and emotional well-being [6].

As the integration of supportive care for patients diagnosed with melanoma and their caregivers into routine clinical practice remains a goal for multidisciplinary care, there is limited research exploring in detail their supportive care needs within Australia. Although research on the supportive care needs of melanoma patients and caregivers has been conducted in the Netherlands [7, 8], the United Kingdom [9], and the United States [10], it’s conceivable that differences between these countries and Australia regarding healthcare systems, access to melanoma treatments, and availability of psychosocial supports exist. Thus, to set a research agenda to address unmet needs in Australia, research from Australian melanoma patients and caregivers should be conducted. Regarding Australian research, one systematic review [11] and six studies were identified by the authors. Of these studies, four focused on melanoma patients [12–16], one on melanoma caregivers [17], and one included both groups [18]. Most of these studies included patients diagnosed with early-stage (American Joint Committee on Cancer stage 0–II [19]) melanoma [12–16], with the most recent study surveying patients between 2010 and 2013 [12, 13], indicating a need for current data. There is only one Australian qualitative study which explored the experiences of advanced-stage (stage III–IV) patients treated with immunotherapy, which has identified some needs [18]. Additionally, there are two qualitative studies investigating the experiences of caregivers of advanced-stage patients [17, 18]. Therefore, the present study contributed to the current Australian literature in several ways: (1) it provides more contemporary data on the supportive care needs of early-stage patients, (2) it has been specifically designed to assess supportive care needs of advanced-stage patients and caregivers, which will contribute to the available literature describing their experiences, and (3) by recruiting both early and advanced-stage patients, this study explored variation of the needs between stages, which is helpful for service and intervention design.

Accordingly, this study aimed to (1) investigate the prevalence of unmet needs, psychosocial outcomes, service utilisation, and service preferences of early-stage and advanced-stage melanoma patients and their caregivers, (2) investigate whether differences in unmet needs and psychosocial outcomes exist between early-stage and advanced-stage melanoma patients, which may necessitate different intervention approaches and priorities and (3) identify variables associated with reporting unmet needs in early-stage and advanced-stage melanoma patients and their caregivers.

## Methods

### Study design

The present study was conducted using a cross-sectional design using a self-reported quantitative survey, available online or in-paper. Respondents were also invited to participate in a semi-structured interview, aiming to explore the lived experiences and perceived needs of melanoma patients and caregivers. To ensure both quantitative and qualitative results are described in sufficient detail, this article will report on the quantitative results while a subsequent publication will detail the qualitative findings. The Strengthening the Reporting of Observational Studies in Epidemiology (STROBE) [20, 21] checklist was used to guide the reporting of this study.

### Setting and participants

This study utilised convenience sampling, recruiting melanoma patients and caregivers from three Australian study sites: (1) two clinics (Sydney Melanoma and Surgical Oncology, and Melanoma Dermatology) which are affiliated with Melanoma Institute Australia, located in Sydney, New South Wales; (2) Melanoma Patients Australia, a national non-profit organisation supporting Australians affected by melanoma; and (3) the medical oncology clinic of Princess Alexandra Hospital, located in Woolloongabba, Queensland.

All participants were aged 18 years or older, with sufficient command of the English language to complete study activities. Melanoma patients had a previous diagnosis of stage 0–II (early-stage) or stage III–IV (advanced-stage) melanoma and were currently receiving treatment or follow-up care regarding their diagnosis or had completed this care in the previous 2 years. Melanoma patients were excluded if they had a diagnosis of a cancer other than skin cancer within the past 5 years. Melanoma caregivers were self-identified as a partner, family member or friend of an individual who satisfied the above inclusion criteria as a melanoma patient. Caregivers were excluded if they provided formal or professional support. Recruitment was completed utilising several methods. An invitation email to participate in the study was distributed via Melanoma Institute Australia and Melanoma Patient Australia emailing lists and social media in August–November 2020. Furthermore, melanoma practitioners at participating clinics approached eligible patients and caregivers, introducing the study, and providing a printed study advertisement with a QR code and/or study package, containing the printed study materials and reply-paid envelope. Finally, after completing the survey, patients/caregivers were prompted to ask their respective caregivers/patients if they would also like to participate in the study, and if so, provided their email address for the researchers to send a study invitation.

### Study outcomes

*Supportive care needs of melanoma patients* were assessed using two measures: the Supportive Care Needs Survey 34-item short-form (SCNS-SF34) [22, 23] and the accompanying Supportive Care Needs Survey Melanoma module (SCNS-M12) [23, 24]. The SCNS-SF34 assesses cancer-related needs (i.e., satisfied, low need, moderate need, and high need) across five domains of need: psychological, health system and informational, physical and daily living, patient care and support, and sexuality. The SCNS-M12 includes a further 12 items assessing melanoma-specific needs. Patients respond to both the SCNS-SF34 and SCNS-M12 items using a 5-point Likert scale, where higher scores indicate higher reported needs.

As no subscales for the SCNS-M12 are available in the literature, exploratory factor analysis was conducted to assess whether items could be combined into appropriate subscales. Both the Kaiser–Meyer–Olkin measure of sample adequacy and Bartlett test of sphericity were calculated to test the appropriateness of the sample size, with subscales identified using eigenvalues > 1. Using this method, the twelve items of the SCNS-M12 were condensed into needs related to two domains: melanoma treatment outcomes (items 1, 2 and 12) and melanoma-related information (items 3–11).

*Supportive care needs in melanoma caregivers* were measured using the 45-item Supportive Care Needs Survey—Partners and Caregivers module (SCNS-P&C) [25] across four domains of need: healthcare service, psychological and emotional, work and social, and information. The SCNS-P&C follows the same response structure and scoring as the SCNS-SF34.

*Quality of life in melanoma patients and caregivers* was measured using the European Organisation for Research and Treatment of Cancer’s Core Quality of Life Questionnaire Version 3.0 (QLQ-C30) [26]. The QLQ-C30 consists of 30 items that form six single item symptom scales (dyspnoea, insomnia, appetite loss, constipation, diarrhoea, and financial difficulties), three multi-item symptom scales (fatigue, nausea and vomiting, and pain), five multi-item functional scales (physical, role, emotional, cognitive, and social), and a multi-item global health status/quality of life scale (referred to as global health status). The QLQ-C30 is measured using a multi-point Likert scale, with higher scores indicating better functioning and global health status, or worse symptomology, on their respective scales. The QLQ-C30 is the most commonly used QoL measure in melanoma [27].

Furthermore, melanoma patients also completed the Melanoma Concerns Questionnaire (MCQ-28) [28]. The MCQ-28 consists of 28 items examining quality of life issues across four multi-item domains (disease prognosis and acceptance, treatment concerns and future disease risk, supportive care, and care delivery and communication). The MCQ-28 follows the same response structure as the QLQ-C30, with higher scores indicating a better outcome, except for the treatment concerns and future disease risk domain, where higher scores indicate increased concern.

*Fear of cancer recurrence (FCR) in melanoma patients* was measured using the Fear of Cancer Recurrence Inventory 9-item short-form (FCRI-9) [29]. The FCRI-9 assesses severity of fear of cancer recurrence and is measured on a 5-point Likert scale, with higher scores indicating greater FCR severity.

*Depression, anxiety, and stress in melanoma patients and caregivers* was measured using the Depression, Anxiety and Stress 21-item short-form (DASS-21) [30]. The DASS-21 is measured using a 4-point Likert scale, with higher scores indicating greater levels of depression, anxiety, or stress, respectively.

All outcome measures have demonstrated acceptable psychometric properties (i.e., reliability, validity) [22, 25, 29–33].

*Demographic variables, service utilisation, and service preferences* in participants was measured using purpose-designed questions.

### Statistical analysis

Questionnaires were analysed and considered complete if data were available for at least 50% of its scales. A total ‘general’, ‘melanoma-related’ and ‘caregiver’ unmet needs score was calculated by dichotomising and summing whether the patient indicated no need (0) or at least some need (1) for all items of the SCNS-34, SCNS-M12 and SCNS-P&C respectively.

Descriptive statistics were used to report participant demographics, unmet needs, psychosocial outcomes, service utilisation, and service preferences. To assist in the clinical interpretation of these descriptive statistics, the FCRI-9 and DASS-21 were categorised according to clinical cut-off scores available in the literature. As multiple cut-off scores have been suggested in the literature; however, to maximise sensitivity, FCRI-9 scores of < 13 were used to indicate that a melanoma patient reported low FCR, whereas scores of 13–21 indicated moderate FCR [34], and scores of $\ge$ 22 indicated severe FCR [35]. Participant levels of anxiety, stress, and depression scores were categorised as normal, mild, moderate, severe, and extremely severe as outlined in the DASS-21 manual [30]. However, FCRI-9 and DASS-21 scores were only treated categorically for the purposes of descriptive analysis, and were treated continuously in all other analyses. It should be noted that due to an administrative error, a total of 11 early-stage and 20 advanced-stage melanoma patients were not provided the FCRI-9 questionnaire to complete. This error was addressed using the statistical analysis methods outlined below.

To analyse differences between early-stage and advanced-stage melanoma patients regarding unmet needs and psychosocial outcomes, independent t-tests were planned; however, due to non-normally distributed data, Mann–Whitney U tests was conducted on all unmet needs and psychosocial outcomes except for severity of FCR, which was normally distributed. To identify variables significantly associated with reporting at least one unmet need in melanoma patients, stepwise multivariable logistic regression was used. A total of four logistic regression models were created to predict: (1) general unmet needs in early-stage patients; (2) melanoma-related unmet needs in early-stage patients; (3) general unmet needs in advanced-stage patients; and (4) melanoma-related unmet needs in advanced-stage patients, with multiple imputation used to handle missing values. Suitable demographic and psychosocial variables for inclusion in the multivariable models were identified through univariable logistic regression at $\alpha$ = 0.25. Although planned, logistic regression analysis was not conducted in caregivers due to a small proportion of caregivers reporting no unmet needs (n = 4, 11%). Results were reported as odds ratios (OR) and their associated 95% confidence intervals (95% CI), with a two-sided* p* $\le$ 0.05 indicating statistical significance. Analysis was conducted using SPSS [36].

## Results

A total of 156 patients and 37 caregivers completed the survey between August 2020 and December 2021. Of the 156 patients, 56 self-identified as early-stage patients, and 100 as advanced-stage patients. The demographic characteristics of patients and caregivers is outlined in Table 1.

**Table 1:** Participant characteristics

Variable	Early-stage (n = 56)*	Advanced-stage (n = 100)*	Caregivers (n = 37)*
Age (mean, SD)	56 (14)	59 (12)	55 (12)
Gender (n, %)			
Male	12 (21%)	46 (46%)	5 (14%)
Female	43 (77%)	54 (54%)	32 (86%)
Family status (n, %)			
Partnered/married/defacto	43 (77%)	81 (81%)	37 (100%)
Single/divorced/widowed	13 (23%)	19 (19%)	0 (0%)
Education (n, %)			
High school or lower	19 (34%)	48 (48%)	18 (49%)
University undergraduate degree	24 (43%)	24 (24%)	12 (32%)
University postgraduate degree	12 (21%)	28 (28%)	7 (19%)
Residence (n, %)			
Urban	43 (77%)	73 (73%)	32 (86%)
Rural/remote	13 (23%)	27 (27%)	5 (14%)
Annual family income (n, %)			
≤ $50,000 AUD	8 (14%)	21 (21%)	7 (19%)
$50,001–100,000 AUD	12 (21%)	24 (24%)	6 (16%)
$100,001–200,000 AUD	17 (30%)	31 (31%)	11 (30%)
> $200,000 AUD	12 (21%)	18 (18%)	7 (19%)
Prefer not to answer	6 (11%)	6 (6%)	5 (14%)
Previous treatment^a^ (n, %)			
Surgery	44 (79%)	81 (81%)	30 (81%)
Radiation therapy	3 (5%)	28 (28%)	13 (35%)
Targeted therapy	1 (2%)	25 (25%)	14 (38%)
Immunotherapy	2 (4%)	86 (86%)	28 (76%)
Chemotherapy	1 (2%)	3 (3%)	2 (5%)
Other	12 (21%)	9 (9%)	4 (10%)
Disease status (n, %)			
Complete remission	44 (79%)	53 (53%)	11 (30%)
Partial remission	1 (2%)	14 (14%)	5 (14%)
Stable disease	1 (2%)	12 (12%)	9 (24%)
Progressive disease	1 (2%)	9 (9%)	10 (27%)
I do not know	8 (14%)	11 (11%)	2 (5%)
Relationship to patient (n, %)			
Partner	–	–	29 (78%)
Immediate family member	–	–	8 (22%)
Recruitment method (n, %)			
MIA email/social media	30 (54%)	35 (35%)	8 (22%)
MPA email/Facebook group	4 (7%)	25 (25%)	14 (38%)
PAH melanoma specialist	0 (0%)	28 (28%)	2 (5%)
MIA melanoma specialist	22 (39%)	9 (9%)	3 (8%)
Dyad^b^	0 (0%)	3 (3%)	10 (27%)

*MIA* Melanoma Institute Australia; *MPA* Melanoma Patients Australia; *PAH* Princess Alexandra Hospital; *SD* standard deviation

*Totals for each variable may not equal 56 (early-stage), 100 (advanced-stage), or 37 (caregivers) due to missing values

^a^Percentages may not equal 100% due to categories not being mutually exclusive

^b^Patients recruited through their caregivers, and caregivers recruited through their patients

### Prevalence of unmet needs, psychosocial outcomes, and service utilisation and preferences

Unmet needs were reported by the majority of participants, with 42 (75%) early-stage patients, 83 (83%) advanced-stage patients, and 33 (89%) caregivers reporting at least one unmet need. Both early-stage (n = 31, 55%) and advanced-stage (n = 63, 63%) patients most frequently reported requiring additional support regarding the ‘fear of melanoma spreading’. The most reported unmet need amongst caregivers was ‘getting emotional support for yourself’ (n = 28, 75%). However, when unmet needs were categorised based on level of importance, the item most reported as high importance to participants was ‘to be informed about how and when to check for skin changes’ in early-stage patients (n = 9, 16%), ‘fear of melanoma spreading’ in advanced-stage patients (n = 17, 17%), and ‘working through your feelings about death and dying’ for caregivers (n = 10, 27%). Of the top ten most reported unmet needs, the majority belonged to the psychological domain in early-stage (n = 6, 60%) and advanced-stage (n = 8, 80%) patients, and to the psychological/emotional domain in caregivers (n = 8, 80%). All unmet needs where at least 25% of participants (stratified by group) identified some level of need and the associated domain is reported in Supplementary Table 1.

**Fig. 1:**
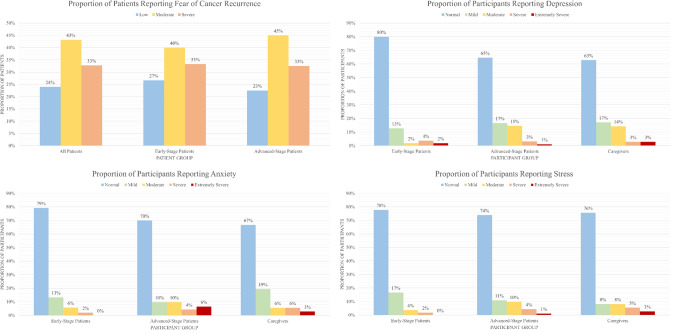
Proportions of participants reporting fear of cancer recurrence, depression, anxiety, and stress

The proportion of patients reporting low, moderate, and severe FCR as well as the proportion of all participants reporting normal-to-extremely severe levels of depression, anxiety and stress are shown below in Fig. 1. In both melanoma patient groups, the majority (> 70%) of participants reported a level of FCR indicative of need for intervention. However, the majority (> 60%) of all participant groups did not report elevated levels of depression, anxiety, and stress. The mean (standard deviation, SD) and median (inter-quartile range, IQR) scores of all subscales of the SCNS-34, SCNS-M12, SCNS-P&C, QLQ-C30, MCQ-28, FCRI-9 and DASS-21 are reported in Supplementary Table 2.

Previous and desired access to various support services is provided in Table 2. Early-stage patients most frequently reported not utilising any supportive care services (n = 21, 46%), followed by utilising a melanoma nurse (n = 10, 18%), and a massage therapist (n = 9, 16%). In advanced-stage patients, a melanoma nurse was also the most utilised (n = 65, 65%), followed by a physiotherapist (n = 40, 40%). Finally, caregivers utilised a psychologist the most (n = 13, 35%), followed by a massage therapist (n = 12, 32%). In regard to support services that participants would have preferred to have access to, all participant groups reported a desire to see a psychologist (> 25%). Advanced-stage patients also desired to see an exercise physiologist (n = 28, 28%) and melanoma nurse (n = 25, 25%). Caregivers also reported a desire to see a melanoma nurse (n = 11, 30%).

**Table 2:** Service utilisation and preferences of support services across participant groups

Support service	Participant group	Accessed n (%)	Desired to access n (%)
Acupuncture	Early-stage patients	5 (9%)	4 (7%)
	Advanced-stage patients	6 (6%)	6 (6%)
	Caregivers	2 (5%)	3 (8%)
Cancer nurse	Early-stage patients	10 (18%)	5 (9%)
	Advanced-stage patients	**65 (65%)**	**25 (25%)**
	Caregivers	8 (22%)	**11 (30%)**
Chinese medicine practitioner	Early-stage patients	1 (2%)	1 (2%)
	Advanced-stage patients	5 (5%)	5 (5%)
	Caregivers	2 (5%)	1 (3%)
Chiropractor	Early-stage patients	5 (9%)	2 (4%)
	Advanced-stage patients	14 (14%)	6 (6%)
	Caregivers	2 (5%)	1 (3%)
Dietician	Early-stage patients	3 (5%)	7 (13%)
	Advanced-stage patients	**30 (30%)**	23 (23%)
	Caregivers	4 (11%)	2 (5%)
Exercise physiologist	Early-stage patients	1 (2%)	8 (14%)
	Advanced-stage patients	19 (19%)	**28 (28%)**
	Caregivers	1 (3%)	1 (3%)
Genetic counsellor	Early-stage patients	2 (4%)	11 (20%)
	Advanced-stage patients	6 (6%)	11 (11%)
	Caregivers	4 (11%)	6 (16%)
Massage therapist	Early-stage patients	9 (16%)	9 (16%)
	Advanced-stage patients	19 (19%)	19 (19%)
	Caregivers	**12 (32%)**	8 (22%)
Nutritionist	Early-stage patients	3 (5%)	12 (21%)
	Advanced-stage patients	4 (4%)	**25 (25%)**
	Caregivers	2 (5%)	1 (3%)
Occupational therapist	Early-stage patients	1 (2%)	0
	Advanced-stage patients	15 (15%)	7 (7%)
	Caregivers	1 (3%)	0
Osteopath	Early-stage patients	2 (4%)	3 (5%)
	Advanced-stage patients	7 (7%)	9 (9%)
	Caregivers	3 (8%)	0
Physiotherapist	Early-stage patients	7 (13%)	4 (7%)
	Advanced-stage patients	**40 (40%)**	21 (21%)
	Caregivers	6 (16%)	2 (5%)
Psychiatrist	Early-stage patients	2 (4%)	0
	Advanced-stage patients	10 (10%)	11 (11%)
	Caregivers	1 (3%)	1 (5%)
Psychologist	Early-stage patients	8 (14%)	**16 (29%)**
	Advanced-stage patients	**37 (37%)**	**26 (26%)**
	Caregivers	**13 (35%)**	**15 (41%)**
Podiatrist	Early-stage patients	4 (7%)	3 (5%)
	Advanced-stage patients	7 (7%)	10 (10%)
	Caregivers	7 (19%)	0
Reflexologist	Early-stage patients	0	3 (5%)
	Advanced-stage patients	3 (3%)	7 (7%)
	Caregivers	0	1 (3%)
Social worker	Early-stage patients	0	1 (2%)
	Advanced-stage patients	9 (9%)	9 (9%)
	Caregivers	4 (11%)	6 (16%)
Speech pathologist	Early-stage patients	0	0
	Advanced-stage patients	5 (5%)	1 (1%)
	Caregivers	0	0
Spiritual guidance	Early-stage patients	2 (4%)	2 (4%)
	Advanced-stage patients	4 (4%)	7 (7%)
	Caregivers	2 (5%)	0
Other	Early-stage patients	8 (14%)	3 (5%)
	Advanced-stage patients	4 (4%)	4 (4%)
	Caregivers	5 (14%)	3 (8%)

Bold, responses where over 25% of participants had access/desired access to this service

### Comparisons between early-stage and advanced-stage melanoma patients

The results of several Mann–Whitney U tests to detect differences in unmet needs and psychosocial outcomes between early and advanced-stage melanoma patients are reported below in Table 3.

**Table 3:** Mann–Whitney U test results for differences between early-stage and advanced-stage melanoma patients

Variable	Participant stage	n	Mean rank	U	Z	*p*
SCNS-36						
Psychological needs	Early-stage	51	64.4	1959.5	− 2.078	0.038*
	Advanced-stage	97	79.8			
Health system & informational needs	Early-stage	53	68.5	2198.5	− 1.465	0.143
	Advanced-stage	97	79.3			
Physical & daily living needs	Early-stage	54	56.4	1561.0	− 3.937	< 0.001*
	Advanced-stage	94	84.9			
Patient care & supportive needs	Early-stage	55	72.9	2468.5	− 0.772	0.440
	Advanced-stage	97	78.6			
Sexuality needs	Early-stage	56	62.1	1881.0	− 3.255	0.001*
	Advanced-stage	96	84.9			
Total general unmet needs	Early-stage	56	72.2	2444.5	− 1.321	0.187
	Advanced-stage	100	82.1			
SCNS-M12						
Melanoma treatment outcome needs	Early-stage	53	67.4	2140.5	− 1.542	0.123
	Advanced-stage	95	78.5			
Melanoma information needs	Early-stage	52	71.0	2312.5	− 0.540	0.589
	Advanced-stage	94	74.9			
Total melanoma-related unmet needs	Early-stage	56	81.0	2569.0	− 0.536	0.592
	Advanced-stage	100	77.1			
QLQ-C30						
Global health status	Early-stage	56	84.0	2491.5	− 1.153	0.249
	Advanced-stage	100	75.4			
Physical functioning	Early-stage	56	88.9	2162.0	− 2.379	0.017*
	Advanced-stage	99	71.8			
Role functioning	Early-stage	56	89.8	2170.0	− 2.510	0.012*
	Advanced-stage	100	72.2			
Emotional functioning	Early-stage	56	88.3	2250.5	− 2.054	0.040*
	Advanced-stage	100	73.0			
Cognitive functioning	Early-stage	56	90.5	2127.5	− 2.622	0.009*
	Advanced-stage	100	71.8			
Social functioning	Early-stage	56	91.7	2062.5	− 2.905	0.004*
	Advanced-stage	100	71.1			
Fatigue	Early-stage	56	63.0	1934.0	− 3.247	0.001*
	Advanced-stage	100	87.2			
Nausea & vomiting	Early-stage	56	69.5	2294.0	− 2.515	0.012*
	Advanced-stage	100	83.6			
Pain	Early-stage	56	71.5	2406.0	− 1.543	0.123
	Advanced-stage	100	82.4			
Dyspnoea	Early-stage	56	66.4	2123.5	− 3.104	0.002*
	Advanced-stage	100	85.3			
Insomnia	Early-stage	56	69.2	2277.5	− 1.936	0.053
	Advanced-stage	99	83.0			
Appetite loss	Early-stage	55	71.6	2396.5	− 1.812	0.070
	Advanced-stage	100	81.5			
Constipation	Early-stage	56	73.8	2537.0	− 1.108	0.268
	Advanced-stage	99	80.4			
Diarrhoea	Early-stage	55	73.0	2474.5	− 1.416	0.157
	Advanced-stage	100	80.8			
Financial struggles	Early-stage	56	69.8	2313.0	− 2.142	0.032*
	Advanced-stage	100	83.4			
MCQ-28						
Disease prognosis & acceptance	Early-stage	56	82.5	2520.0	− 0.943	0.346
	Advanced-stage	99	75.5			
Treatment concerns & future disease risk	Early-stage	56	86.3	2306.0	− 1.738	0.082
	Advanced-stage	99	73.3			
Supportive care	Early-stage	55	58.5	1675.5	− 4.052	< 0.001*
	Advanced-stage	100	88.8			
Care delivery & communication	Early-stage	54	55.3	1503.0	− 4.595	< 0.001*
	Advanced-stage	100	89.5			
DASS-21						
Depression	Early-stage	55	67.6	2179.5	− 1.804	0.071
	Advanced-stage	96	80.8			
Anxiety	Early-stage	53	65.9	2060.0	− 1.685	0.092
	Advanced-stage	93	77.9			
Stress	Early-stage	54	69.3	2259.5	− 0.916	0.360
	Advanced-stage	92	75.9			

*DASS-21* Depression, Anxiety and Stress 21-item short-form; *MCQ-12*, Melanoma Concerns Questionnaire; *QLQ-C30* European Organisation for Research and Treatment of Cancer’s Core Quality of Life Questionnaire; *SCNS-34* Supportive Care Needs Survey 34-item short-form; *SCNS-M12* Supportive Care Needs Survey Melanoma Module

*Statistically significant at $\alpha$ = 0.05

Advanced-stage melanoma patients reported significantly more unmet psychological needs (*U* = 1959.50,* p* < 0.05), physical and daily living needs (*U* = 1561.00, *p* < 0.001), and sexual needs (*U* = 1881.00, *p* < 0.001). Early-stage and advanced-stage melanoma patients did not differ in relation to health system and informational needs, patient care and support needs, melanoma treatment outcome needs, melanoma-specific informational needs, or total unmet needs.

Although no significant difference was evident regarding overall QoL, advanced-stage melanoma patients reported significantly worse functioning across all functioning scales (*p* < 0.05), as well as reporting higher levels of fatigue (*U* = 1934.00, *p* < 0.001), nausea and vomiting (*U* = 2294.00, *p* < 0.05), dyspnoea (*U* = 2123.50, *p* < 0.01), and financial difficulties (*U* = 2313.00, *p* < 0.05). Furthermore, no difference was evident regarding acceptance of disease prognosis or treatment concerns and future risk. However, advanced-stage melanoma patients reported significantly better perceptions of supportive care (*U* = 1675.50, *p* < 0.001) and care delivery and communication (*U* = 1503.00, *p* < 0.001) when compared to early-stage patients. Early and advanced-stage melanoma patients did not differ in relation to FCR severity (t_123_ = − 0.25, *p* = 0.803), depression, anxiety, or stress (Table 3).

### Variables associated with unmet needs

Univariable logistic regression results are reported in Supplementary Tables 3 and 4. The four stepwise multivariable logistic regression models are presented in Table 4.

**Table 4:** Stepwise multivariable logistic regression results for the prediction of general unmet needs in melanoma patients

Model^a^	Odds ratio (95% CI)	*p* value
Model 1: early-stage patients, general unmet needs^b^		
Income		0.090
≤ $50,000 AUD	Reference	–
$50,001–100,000 AUD	0.04 (0.01, 0.96)	0.047*
$100,001–200,000 AUD	0.76 (0.04, 14.05)	0.850
> $200,000 AUD	0.08 (0.01, 2.40)	0.145
Disease prognosis & acceptance	0.80 (0.63, 1.01)	0.063
Fear of cancer recurrence	1.20 (1.02, 1.39)	0.026*
Anxiety	2.45 (1.17, 5.10)	0.017*
Model 2: early-stage patients, melanoma-related unmet needs^c^		
Care delivery & communication	0.66 (0.44, 0.98)	0.043*
Anxiety	1.87 (1.21, 2.89)	0.005*
Model 3: advanced-stage patients, general unmet needs^d^		
Age	0.93 (0.86, 1.00)	0.043*
Family status (partnered)	0.90 (0.01, 1.11)	0.061
Global health status	0.94 (0.89, 0.99)	0.039*
Fear of cancer recurrence	1.17 (0.99, 1.39)	0.065
Stress	1.65 (1.07, 2.52)	0.022*
Model 4: advanced-stage patients, melanoma-related unmet needs^e^		
Global health status	0.96 (0.93, 0.99)	0.022*
Disease risk & future concerns	1.16 (1.01, 1.33)	0.037*
Supportive care	0.82 (0.70, 0.96)	0.012
Anxiety	1.33 (1.00, 1.76)	0.050*

*CI* confidence interval

*Statistically significant at $\alpha$ = 0.05

^a^All stepwise models included the following variables: global health status, disease prognosis & acceptance, supportive care, care delivery & communication, fear of cancer recurrence, anxiety, and stress

^b^Additional variables included in stepwise model: age, residence, income, depression. Overall model significance: χ^2^ = 34.62, df = 6, p ≤ 0.001. Nagelkerke R: 0.661. Hosmer–Lemeshow Test: χ^2^ = 7.48, df = 7, p = 0.381. Classification Rate: 85.7%. AUC: 0.942, p ≤ 0.001

^c^Additional variables included in stepwise model: none. Overall model significance: χ^2^ = 16.66, df = 2, p ≤ 0.001. Nagelkerke R: 0.351. Hosmer–Lemeshow Test: χ^2^ = 7.24, df = 8, p = 0.511. Classification Rate: 76.8%. AUC: 0.797, p ≤ 0.001

^d^Additional variables included in stepwise model: age, gender, family status, residence, income, depression. Overall model significance: χ^2^ = 48.76, df = 5, p ≤ 0.001. Nagelkerke R: 0.632. Hosmer–Lemeshow Test: χ^2^ = 4.80, df = 8, p = 0.779. Classification Rate: 89.0%. AUC: 0.938, p ≤ 0.001

^e^Additional variables included in stepwise model significance: gender. Overall model significance: χ^2^ = 49.08, df = 4, p ≤ 0.001. Nagelkerke R: 0.530. Hosmer–Lemeshow Test: χ^2^ = 9.20, df = 8, p = 0.325. Classification Rate: 84.0%. AUC: 0.880, p ≤ 0.001

In early-stage melanoma patients, increased FCR was significantly associated with an increased odds of reporting a general unmet need (OR 1.87; 95% CI 1.21, 2.89; p < 0.01), whereas improved clinical care delivery and clinician communication was significantly associated with a decreased odds of reporting a melanoma-related unmet need (OR 1.20; 95% CI 1.02, 1.39; p < 0.05). Furthermore, increased anxiety was significantly associated with both an increased odds of reporting a general (OR 2.45; 95% CI 1.17, 5.10; p < 0.05) and melanoma-related (OR 1.87; 95% CI 1.21, 2.89; p < 0.01) unmet need.

In advanced-stage melanoma patients, younger age was significantly associated with a decreased odds of reporting a general unmet need (OR 0.93; 95% CI 0.86, 0.99; p < 0.05), whilst stress was significantly associated with an increased odds (OR 1.65; 95% CI 1.08, 2.52; p < 0.05). A significantly higher odds of reporting a melanoma-related unmet need was associated with increased perceived disease risk and future concerns (OR 1.16; 95% CI 1.01, 1.33; p < 0.05) and anxiety (OR 1.33; 95% CI 1.00, 1.76; p < 0.05), whereas supportive care received was associated with decreased odds (OR 0.82; 95% CI 0.70, 0.96; p < 0.05). Increased overall QoL was significantly associated with a decreased odds of reporting a general (OR 0.94; 95% CI 0.89, 0.99; p < 0.05) and melanoma-related (OR 0.96; 95% CI 0.93, 0.99; p < 0.05) unmet needs.

All four models reported good predictive power and were a good fit for the data (Table 4). Classification rates ranged from 76.8 to 89.0%, and area under the curve ranged from 79.7 to 94.2%. Variation in unmet needs predicted by each model ranged from 35.1 to 66.1%.

## Discussion

To the authors’ knowledge, this is the first Australian study to investigate the prevalence of unmet supportive care needs, psychosocial outcomes, service utilisation, and service preferences of early-stage and advanced-stage melanoma patients, their caregivers, and identified potential predictors of reporting an unmet supportive care need in the era of effective drug therapy. Over 75% of patients and caregivers, regardless of disease stage, reported at least one unmet supportive care need. Of these, the most common were related to psychological and emotional well-being. These results are consistent with previous research in Australian cancer patients [14, 37, 38], although the present study reported a slightly higher number of unmet needs in melanoma patients. Furthermore, this study identified that levels of FCR were uniform across both early-stage and advanced-stage patients, indicating that FCR is a prevalent and unaddressed issue equally effecting melanoma patients of all stages.

Of the support services investigated, access to a psychologist was desired by a substantial proportion of melanoma patients and caregivers. This is unsurprising given that a diagnosis and treatment of melanoma, or caring for someone with melanoma, has a significant impact on mental health and well-being [12, 39], with psycho-oncological support generally underfunded by the Australian Federal Government [40]. Advanced melanoma patients also desired access to both an exercise physiologist and nutritionist, which is unsurprising given that these patients are more likely to experience physical functioning impairments and fatigue [5, 41], with these services addressing the physical sequalae of the disease. A substantial proportion of advanced melanoma patients and caregivers also desired access to a cancer nurse. A 2022 landmark Australian independent publication, the *State of the Nation—A Report into Melanoma* [5] identified melanoma nurses as a pivotal member of the healthcare team that can provide additional support to patients, with innovative nurse-led supportive care interventions currently being trialled in the advanced melanoma space [42]. Furthermore, the Australian Department of Health and Aged Care has promised $14.8 million AUD to fund 30 specialist melanoma nurses to further address this need [43]. Thus, this unmet need may be addressed in the coming years.

Although disease stage was not related to whether a patient reported a general or melanoma-related unmet need, advanced-stage patients reported significantly more psychological, physical and daily living, and sexuality needs. This is likely a result of the various adverse events associated with advanced melanoma drug therapy and functional symptoms associated with metastases [5, 44]. Advanced melanoma patients reported significantly worse functioning across all functional scales, fatigue, nausea and vomiting, dyspnoea, and financial struggles, which could be attributed to both the physical and psychological impact of advanced melanoma diagnosis and treatment [41, 44]. These results highlight that even though early-stage and advanced-stage melanoma patients report the same needs, advanced-stage patients report potentially more needs due to the impact of the advanced disease, highlighting the importance of supportive care interventions for advanced-stage patients. To date, a majority of supportive care interventions in the melanoma space have focused on early-stage patients [44–48], with interventions in the advanced space beginning to emerge [42, 49, 50]. Furthermore, the implementation of these interventions into routine care is severely lacking, with few implementation studies existing in the melanoma space [46]. Lastly, these results highlight the opportunity to deliver simple and effective educational support to early-stage patients, as the need to be informed regarding how and when to conduct skin checks was the most reported unmet need of high importance within this group.

Several significant associations of general and melanoma-related unmet needs in early-stage and advanced-stage melanoma patients were identified. It is not surprising that both anxiety and FCR were related to unmet needs in both early-stage and advanced-stage patients, as the most reported unmet needs were related to psychological and emotional well-being. Thus, it is evident that current service models are not addressing the psychological and emotional well-being of melanoma patients. This is consistent with the *State of the Nation—A Report into Melanoma* [5], as over 30% of melanoma patients reported anxiety, yet 40% reported this topic not being discussed with their healthcare team. Furthermore, QoL was related to unmet needs in advanced-stage patients, which again in unsurprising given the functional impairments that may result from current treatments [41, 44]. Based on the variables significantly associated with unmet needs identified in this study, it is imperative that clinician-patient communication regarding physical and emotional QoL be improved. This can be facilitated by the implementation of routine patient-reported outcome measures to screen melanoma patients prior to follow-up consultations to encourage this conversation between clinicians and patients [42, 51], or through the widescale adoption of communication-based skills training for clinicians to increase their confidence in discussing supportive care needs with patients [52, 53]. The implementation of these strategies also represents an important step toward the integration of supportive care into routine clinical practice and referral pathways, as although well-established protocols exist regarding the treatment of melanoma with accompanying recommendations for treatment pathways and multidisciplinary teams [54], access to supportive care services (i.e., melanoma nurses, psychologists) are limited at present within Australia [5]. Finally, although caregivers reported substantial unmet needs, future research should aim to replicate these findings with a larger sample size to both increase confidence in these results and identify potential predictors.

These results should be interpreted in the context of their limitations. Due to the nature of the convenience sampling method used, the extent to which selection bias may be impacting these results is unknown. Additionally, due to restrictions implemented as a result of the COVID-19 pandemic within Australia throughout the recruitment period, the convenience sampling method used resulted in a lower-than-expected sample size. Participants in this study reported lower-than-average rates of depression and anxiety compared to the Australian general population [55], which is likely a result of selection bias. Females were also over-represented in both the early-stage melanoma patients and caregiver groups, as the age standardised incidence rate for melanoma in Australia is 50% higher in males compared to females [3]. Furthermore, due to the testing of multiple testing of hypotheses, the odds of a false positive finding is increased, thus the results should be interpreted with caution and replication with larger sample sizes is warranted. Lastly, participants were not asked whether they had accessed support from other patients and caregivers, so the extent to which peer support was utilised amongst these participants is unknown.

Despite these weaknesses, this study has several strengths. The first of these is the use of psychometrically validated outcome measures to ensure results are accurate and reliable. This study also included melanoma patients across all stages of the disease as well as their caregivers, ensuring results can be used to guide service planning and provision based on the unique needs of each group. To the authors knowledge, this study was also the first to investigate which support services were both previously utilised and desired by melanoma patients and caregivers, to further assist in future resource development and provision.

These results assist in providing guidance regarding the design and implementation of supportive care interventions through identifying the most substantial unmet supportive care needs in melanoma patients and caregivers. Understanding the prevalence of unmet needs, desired services, and predictors of unmet needs is a crucial first step in designing, trialling, and implementing supportive care interventions and clinical service prioritisation, such as clinical psychology services incorporating patient screening and stepped-care models [46] and patient-reported outcome measures [51], to improve the well-being of melanoma patients and their caregivers.

### Supplementary Information

Below is the link to the electronic supplementary material.

Supplementary file1 (DOCX 42 KB)

## Data Availability

Study data is not available for sharing, as participants did not consent to data sharing.
