# Supplementary material for: Supportive care needs in Australian melanoma patients and caregivers: results from a quantitative cross-sectional survey
Source: Qual Life Res. 2023 Jul 31;32(12):3531–45. doi: 10.1007/s11136-023-03492-0 (PMC10624748; doi:10.1007/s11136-023-03492-0)
Supplement: Supplementary file 1 — Supplementary file1 (DOCX 42 KB) [file 11136_2023_3492_MOESM1_ESM.docx]

# Supplementary Material

Supplementary Table 1. Most Common Unmet Needs and Domains Across Participant Groups

| **Participants Diagnosed with Early-Stage Melanoma** | | | |
| --- | --- | --- | --- |
| Unmet Need | Percent (%) Reporting Need | Percent (%) Reporting as Low / Moderate / High Need | Domain |
| 9. Fears about melanoma spreading | 55 | 22 / 20 / 13 | Psychological |
| 6. Anxiety | 48 | 26 / 15 / 7 | Psychological |
| 11. Uncertainty about the future | 45 | 18 / 20 / 7 | Psychological |
| MM3. More information about the risk of recurrence of melanoma | 42 | 14 / 14 / 14 | Melanoma-Specific Information |
| 17. Concerns about the worries of those close to you | 40 | 20 / 18 / 2 | Psychological |
| 12. Learning to feel in control of your situation | 37 | 24 / 7 / 6 | Psychological |
| MM9. To be informed about how and when to check for skin changes | 37 | 13 / 8 / 16 | Melanoma-Specific Information |
| 10. Worry that the results of treatment are beyond your control | 36 | 19 / 11 / 6 | Psychological |
| MM1. Skin soreness | 35 | 22 / 11 / 2 | Melanoma Treatment Outcome |
| MM8. To be informed about things you can do for skin protection | 34 | 16 / 11 / 7 | Melanoma-Specific Information |
| 14. Feelings about death and dying | 33 | 18 / 11 / 4 | Psychological |
| 25. Being given explanations of those tests for which you would like explanations | 33 | 13 / 16 / 4 | Health System/Informational |
| 34. Having one member of hospital staff with whom you can talk to about all aspects of your condition, treatment and follow-up | 33 | 15 / 9 / 9 | Health System/Informational |
| 7. Feeling down or depressed | 32 | 15 / 11 / 6 | Psychological |
| 24. Being given written information about aspects of managing your illness and side-effects at home | 31 | 9 / 11 / 11 | Health System/Informational |
| MM7. More information about possible outcomes when melanoma has spread from the skin | 30 | 18 / 5 / 7 | Melanoma-Specific Information |
| MM10. Access to a second opinion about your condition if you want one | 30 | 9 / 7 / 14 | Melanoma-Specific Information |
| 18. More choice about which melanoma specialists you see | 29 | 11 / 9 / 9 | Patient Care/Support |
| 29. Being informed about things you can do to help yourself get well | 29 | 11 / 9 / 9 | Health System/Informational |
| 30. Having access to professional counselling if you, family, or friends need it | 29 | 11 / 7 / 11 | Health System/Informational |
| 8. Feelings of sadness | 28 | 16 / 6 / 6 | Psychological |
| 19. More choice about which hospital you attend | 28 | 9 / 15 / 4 | Patient Care/Support |
| 23. Being given written information about the important aspects of your care | 28 | 2 / 13 / 13 | Health System/Informational |
| 20. Reassurance by medical staff that the way you feel is normal | 27 | 16 / 11 / 0 | Patient Care/Support |
| 28. Being informed about melanoma which is under control or diminishing | 27 | 11 / 5 / 11 | Health System/Informational |
| 32. Being treated like a person and not just another care | 27 | 9 / 9 / 9 | Health System/Informational |
| 13. Keeping a positive outlook | 26 | 17 / 7 / 2 | Psychological |
| **Participants Diagnosed with Advanced Melanoma** | | | |
| Unmet Need | Percent (%) Reporting Need | Percent (%) Reporting as Low / Moderate / High Need | Domain |
| 9. Fears about melanoma spreading | 63 | 30 / 16 / 17 | Psychological |
| 11. Uncertainty about the future | 59 | 27 / 18 / 14 | Psychological |
| 17. Concerns about the worries of those close to you | 57 | 26 / 21 / 10 | Psychological |
| 6. Anxiety | 49 | 19 / 18 / 12 | Psychological |
| 5. Not being able to do things you used to do | 48 | 28 / 11 / 9 | Physical/Daily Living |
| 2. Lack of energy and tiredness | 46 | 26 / 13 / 7 | Physical/Daily Living |
| 8. Feelings of sadness | 43 | 20 / 19 / 4 | Psychological |
| 7. Feeling down or depressed | 42 | 17 / 20 / 5 | Psychological |
| 10. Worry that the results of treatment are beyond your control | 42 | 16 / 12 / 14 | Psychological |
| 12. Learning to feel in control of your situation | 41 | 19 / 12 / 10 | Psychological |
| 30. Having access to professional counselling if you, family, or friends need it | 41 | 15 / 12 / 14 | Health System/Informational |
| MM3. More information about the risk of recurrence of melanoma | 38 | 12 / 18 / 8 | Melanoma-Specific Information |
| 29. Being informed about things you can do to help yourself get well | 36 | 21 / 9 / 6 | Health System/Informational |
| 13. Keeping a positive outlook | 35 | 14 / 12 / 9 | Psychological |
| 14. Feelings about death and dying | 35 | 16 / 13 / 6 | Psychological |
| 1. Pain | 34 | 17 / 13 / 4 | Physical/Daily Living |
| 4. Work around the home | 31 | 20 / 7 / 4 | Physical/Daily Living |
| MM11. More information about the unwanted effects of treatment | 30 | 12 / 14 / 4 | Melanoma-Specific Information |
| 15. Changes in sexual feelings | 29 | 16 / 11 / 2 | Sexuality |
| 16. Changes in your sexual relationships | 29 | 14 / 12 / 3 | Sexuality |
| 34. Having one member of hospital staff with whom you can talk to about all aspects of your condition, treatment and follow-up | 29 | 12 / 8 / 9 | Health System/Informational |
| 3. Feeling unwell a lot of the time | 27 | 15 / 9 / 3 | Physical/Daily Living |
| **Caregivers of People Diagnosed with Melanoma** | | | |
| Unmet Need | Percent (%) Reporting Need | Percent (%) Reporting as Low / Moderate / High Need | Domain |
| 37. Getting emotional support for yourself | 75 | 32 / 24 / 19 | Psychological/Emotional |
| 15. Looking after your own health | 69 | 28 / 22 / 19 | N/A |
| 17. Addressing fears about the patient with melanoma’s physical or mental deterioration | 68 | 22 / 22 / 24 | Healthcare Services |
| 34. Balancing the needs of the patient with melanoma and your own needs | 67 | 35 / 24 / 8 | Psychological/Emotional |
| 32. The impact that melanoma has had on your relationship with the patient with melanoma | 65 | 30 / 19 / 16 | Psychological/Emotional |
| 38. Getting emotional support for your loved ones | 65 | 24 / 30 / 11 | Psychological/Emotional |
| 40. Dealing with others not acknowledging the impact on your life of caring for a patient with melanoma | 65 | 30 / 11 / 24 | Psychological/Emotional |
| 39. Working through your feelings about death and dying | 64 | 32 / 5 / 27 | Psychological/Emotional |
| 33. Understanding the experiences of the patient with melanoma | 62 | 31 / 17 / 14 | Psychological/Emotional |
| 35. Adjusting to changes in the patient with melanoma’s body | 62 | 27 / 16 / 19 | Psychological/Emotional |
| 41. Coping with the patient with melanoma’s recovery not turning out the way you expected | 62 | 24 / 27 / 11 | Psychological/Emotional |
| 14. Reducing stress in the life of the patient with melanoma | 60 | 16 / 22 / 22 | Healthcare Services |
| 3. Accessing information on support services for caregivers or partners | 57 | 27 / 14 / 16 | Informational |
| 31. Managing concerns about the melanoma coming back | 57 | 19 / 30 / 8 | Psychological/Emotional |
| 42. Making decisions about your life in the context of uncertainty | 57 | 27 / 14 / 16 | Psychological/Emotional |
| 19. Caring for the patient with melanoma on a practical level | 55 | 22 / 22 / 11 | N/A |
| 5. Accessing information on what the physical needs of the patient with melanoma are likely to be | 54 | 32 / 3 / 19 | Informational |
| 6. Accessing information about the benefits and side effects of treatments | 54 | 19 / 14 / 14 | Informational |
| 21. Adapting to changes in the working life or usual activities of the patient with melanoma | 54 | 24 / 14 / 16 | Work/Social |
| 22. The impact that caring for the patient with melanoma has had your working life or usual activities | 54 | 27 / 16 / 11 | Work/Social |
| 29. Talking to other people who have cared for someone with melanoma | 52 | 30 / 8 / 14 | Work/Social |
| 10. Having opportunities to discuss your concerns with the doctors | 51 | 24 / 16 / 11 | Healthcare Services |
| 2. Accessing information about the prognosis of the patient with melanoma | 50 | 22 / 14 / 14 | Informational |
| 23. Finding out about financial support and governmental benefits | 47 | 28 / 11 / 8 | Informational |
| 9. Involvement in the care of the patient with melanoma, together with the medical team | 46 | 24 / 14 / 8 | Healthcare Services |
| 16. Obtaining adequate pain control for the patient with melanoma | 46 | 14 / 16 / 16 | Healthcare Services |
| 28. Getting more support from your family | 46 | 27 / 8 / 11 | Work/Social |
| 1. Accessing information relevant to your needs as a caregiver/partner | 44 | 22 / 11 / 11 | Informational |
| 26. Communicating with the patient with melanoma | 44 | 22 / 8 / 14 | Work/Social |
| 4. Accessing information on alternative therapies | 43 | 27 / 5 / 11 | Informational |
| 36. Addressing problems with your sex life | 41 | 14 / 16 / 11 | Psychological/Emotional |
| 45. Having opportunities to participate in decision making about the patient with melanoma’s treatment | 41 | 22 / 8 / 11 | N/A |
| 44. Finding meaning in the patient with melanoma’s illness | 40 | 17 / 17 / 6 | Psychological/Emotional |
| 12. Ensuring there is an ongoing case manager to coordinate services for the patient with melanoma | 38 | 22 / 8 / 8 | Healthcare Services |
| 30. Handling the topic of cancer in social situations or at work | 38 | 27 / 8 / 3 | Work/Social |
| 7. Obtaining the best medical care for the patient with melanoma | 36 | 8 / 11 / 17 | Healthcare Services |
| 24. Getting life and/or travel insurance for the patient with melanoma | 35 | 27 / 3 / 5 | N/A |
| 8. Accessing local health care services when needed | 33 | 19 / 11 / 3 | Healthcare Services |
| 20. Finding more accessible hospital parking | 33 | 14 / 8 / 11 | Healthcare Services |
| 11. Feeling confident that all the doctors consult with each other in order to coordinate care of the patient with melanoma | 29 | 5 / 16 / 8 | Healthcare Services |
| 27. Communicating with the family | 28 | 11 / 11 / 6 | Work/Social |
| 43. Exploring your spiritual beliefs | 28 | 22 / 3 / 3 | Psychological/Emotional |
|  |  |  |  |

Supplementary Table 2. Mean (Standard Deviation) and Median (Inter-Quartile Range) Unmet Need and Psychosocial Outcome Scores Across Participant Groups

| **Participants Diagnosed with Melanoma** | | | | | | | |
| --- | --- | --- | --- | --- | --- | --- | --- |
| Variable |  | n | % | Mean | SD | Median | IQR |
| **SCNS-34** |  |  |  |  |  |  |  |
| Psychological Needs | Early Stage | 51 | 91% | 29.3 | 25.4 | 25.0 | 5.0, 45.0 |
|  | Advanced-Stage | 97 | 97% | 39.5 | 27.9 | 37.5 | 17.5, 61.3 |
| Health System & Informational Needs | Early Stage | 53 | 95% | 27.8 | 28.9 | 18.2 | 4.5, 46.6 |
|  | Advanced-Stage | 97 | 97% | 29.9 | 21.8 | 25.0 | 15.9, 39.8 |
| Physical & Daily Living Needs | Early Stage | 54 | 97% | 14.4 | 18.9 | 7.5 | 0.0, 20.0 |
|  | Advanced-Stage | 94 | 94% | 30.3 | 25.4 | 30.0 | 10.0, 50.0 |
| Patient Care & Support Needs | Early Stage | 55 | 98% | 22.2 | 25.6 | 10.0 | 0.0, 40.0 |
|  | Advanced-Stage | 97 | 97% | 20.9 | 17.1 | 20.0 | 5.0, 30.0 |
| Sexuality Needs | Early Stage | 56 | 100% | 10.9 | 17.4 | 0.0 | 0.0, 16.7 |
|  | Advanced-Stage | 96 | 96% | 23.2 | 25.3 | 16.7 | 0.0, 39.6 |
| Total General Unmet Needs | Early Stage | 56 | 100% | 9.1 | 9.5 | 7.0 | 0.0, 17.8 |
|  | Advanced-Stage | 100 | 100% | 10.9 | 9.6 | 8.5 | 0.0, 19.0 |
| **SCNS-M12** |  |  |  |  |  |  |  |
| Melanoma Treatment Outcome Needs | Early Stage | 53 | 95% | 15.7 | 18.3 | 8.3 | 0.0, 25.0 |
|  | Advanced-Stage | 95 | 95% | 20.9 | 21.0 | 16.7 | 0.0, 33.3 |
| Melanoma-Specific Information Needs | Early Stage | 52 | 93% | 24.4 | 25.1 | 15.3 | 0.7, 41.7 |
|  | Advanced-Stage | 94 | 94% | 23.4 | 19.3 | 22.2 | 8.3, 31.3 |
| Total Melanoma-Related Unmet Needs | Early Stage | 56 | 100% | 3.0 | 3.3 | 2.0 | 0.0, 5.0 |
|  | Advanced-Stage | 100 | 100% | 2.6 | 3.2 | 1.0 | 0.0, 4.0 |
| **QLQ-C30** |  |  |  |  |  |  |  |
| Global Health Status | Early Stage | 56 | 100% | 74.7 | 17.9 | 75.0 | 66.7, 83.3 |
|  | Advanced-Stage | 100 | 100% | 70.8 | 20.2 | 70.8 | 58.3, 83.3 |
| Physical Functioning | Early Stage | 56 | 100% | 90.7 | 14.8 | 100.0 | 86.7, 100.0 |
|  | Advanced-Stage | 99 | 99% | 84.8 | 18.5 | 93.3 | 80.0, 100.0 |
| Role Functioning | Early Stage | 56 | 100% | 85.1 | 23.3 | 100.0 | 66.7, 100.0 |
|  | Advanced-Stage | 100 | 100% | 73.3 | 30.4 | 83.3 | 66.7, 100.0 |
| Emotional Functioning | Early Stage | 56 | 100% | 78.0 | 20.8 | 76.4 | 66.7, 100.0 |
|  | Advanced-Stage | 100 | 100% | 70.3 | 23.8 | 75.0 | 58.3, 91.7 |
| Cognitive Functioning | Early Stage | 56 | 100% | 86.9 | 17.9 | 100.0 | 83.3, 100.0 |
|  | Advanced-Stage | 100 | 100% | 77.0 | 23.3 | 83.3 | 66.7, 100.0 |
| Social Functioning | Early Stage | 56 | 100% | 86.0 | 20.5 | 100.0 | 66.7, 100.0 |
|  | Advanced-Stage | 100 | 100% | 72.7 | 28.9 | 75.0 | 50.0, 100.0 |
| Fatigue | Early Stage | 56 | 100% | 20.4 | 23.0 | 22.2 | 0.0, 33.0 |
|  | Advanced-Stage | 100 | 100% | 33.9 | 26.4 | 33.3 | 11.1, 55.5 |
| Nausea | Early Stage | 56 | 100% | 2.7 | 7.6 | 0.00 | 0.0, 0.0 |
|  | Advanced-Stage | 100 | 100% | 8.3 | 16.3 | 0.0 | 0.0, 16.7 |
| Pain | Early Stage | 56 | 100% | 17.3 | 24.2 | 0.0 | 0.0, 33.0 |
|  | Advanced-Stage | 100 | 100% | 21.7 | 22.9 | 16.7 | 0.0, 33.0 |
| Dyspnoea | Early Stage | 56 | 100% | 6.6 | 17.3 | 0.0 | 0.0, 0.0 |
|  | Advanced-Stage | 100 | 100% | 17.0 | 24.4 | 0.0 | 0.0, 33.3 |
| Insomnia | Early Stage | 56 | 100% | 28.6 | 31.4 | 33.3 | 0.0, 33.0 |
|  | Advanced-Stage | 99 | 99% | 38.4 | 32.1 | 33.3 | 0.0, 67.0 |
| Appetite Loss | Early Stage | 55 | 98% | 6.1 | 15.8 | 0.0 | 0.0, 0.0 |
|  | Advanced-Stage | 100 | 100% | 12.7 | 23.6 | 0.0 | 0.0, 33.0 |
| Constipation | Early Stage | 56 | 100% | 10.7 | 23.0 | 0.0 | 0.0, 0.0 |
|  | Advanced-Stage | 99 | 99% | 15.2 | 26.6 | 0.0 | 0.0, 33.0 |
| Diarrhoea | Early Stage | 55 | 98% | 6.1 | 14.5 | 0.0 | 0.0, 0.0 |
|  | Advanced-Stage | 100 | 100% | 11.0 | 21.2 | 0.0 | 0.0, 33.0 |
| Financial Difficulties | Early Stage | 56 | 100% | 9.5 | 17.7 | 0.0 | 0.0, 25.0 |
|  | Advanced-Stage | 100 | 100% | 21.0 | 31.3 | 0.0 | 0.0, 33.0 |
| **MCQ-28** |  |  |  |  |  |  |  |
| Disease Prognosis & Acceptance | Early Stage | 56 | 100% | 67.9 | 24.4 | 66.7 | 55.6, 88.9 |
|  | Advanced-Stage | 99 | 99% | 65.3 | 22.4 | 66.7 | 55.6, 83.3 |
| Disease Risk & Future Concerns | Early Stage | 56 | 100% | 47.4 | 20.6 | 45.8 | 33.3, 65.6 |
|  | Advanced-Stage | 99 | 99% | 42.2 | 29.3 | 38.9 | 27.8, 55.6 |
| Supportive Care | Early Stage | 55 | 98% | 40.3 | 31.8 | 33.3 | 16.7, 66.7 |
|  | Advanced-Stage | 100 | 100% | 63.4 | 32.1 | 66.7 | 41.7, 100.0 |
| Care Delivery & Communication | Early Stage | 54 | 96% | 42.0 | 28.9 | 50.0 | 16.7, 66.7 |
|  | Advanced-Stage | 100 | 100% | 66.9 | 30.3 | 66.7 | 33.3, 100.0 |
| **Fear of Cancer Recurrence** | Early Stage | 45 | 80% | 17.7 | 7.3 | 18.0 | 11.5, 24.0 |
|  | Advanced-Stage | 80 | 80% | 18.1 | 7.3 | 17.0 | 13.0, 23.8 |
| **DASS-21** |  |  |  |  |  |  |  |
| Depression | Early Stage | 55 | 98% | 2.6 | 3.3 | 1.0 | 0.0, 4.0 |
|  | Advanced-Stage | 96 | 96% | 3.5 | 3.4 | 3.0 | 1.0, 5.8 |
| Anxiety | Early Stage | 53 | 95% | 1.9 | 2.2 | 1.0 | 0.0, 3.0 |
|  | Advanced-Stage | 93 | 93% | 3.0 | 3.5 | 2.0 | 0.0, 4.0 |
| Stress | Early Stage | 54 | 96% | 4.3 | 3.6 | 4.0 | 1.0, 7.0 |
|  | Advanced-Stage | 92 | 92% | 5.0 | 4.3 | 4.0 | 1.3, 8.8 |
| **Caregivers of People Diagnosed with Melanoma** | | | | | | | |
| Variable |  | n | % | Mean | SD | Median | IQR |
| **SCNS-P&C** |  |  |  |  |  |  |  |
| Health Care Service Needs |  | 36 | 97% | 39.7 | 24.3 | 38.8 | 23.1, 55.0 |
| Psychological and Emotional needs |  | 34 | 92% | 46.4 | 22.8 | 48.2 | 35.7, 66.5 |
| Work and Social Needs |  | 36 | 97% | 35.5 | 20.9 | 37.5 | 21.4, 53.6 |
| Informational Needs |  | 36 | 97% | 38.3 | 25.6 | 41.1 | 18.8, 52.7 |
| Total Caregiver Unmet Needs |  | 37 | 100% | 21.4 | 13.2 | 21.0 | 9.0, 32.5 |
| **QLQ-C30** |  |  |  |  |  |  |  |
| Global Health Status |  | 37 | 100% | 68.0 | 21.1 | 66.7 | 58.3, 83.3 |
| Physical Functioning |  | 37 | 100% | 91.2 | 14.5 | 100.0 | 86.7, 100.0 |
| Role Functioning |  | 37 | 100% | 67.1 | 35.0 | 83.3 | 33.3, 100.0 |
| Emotional Functioning |  | 37 | 100% | 59.0 | 27.2 | 58.3 | 41.7, 83.3 |
| Cognitive Functioning |  | 37 | 100% | 71.2 | 28.8 | 83.3 | 66.7, 100.0 |
| Social Functioning |  | 37 | 100% | 77.0 | 30.0 | 83.3 | 66.7, 100.0 |
| Fatigue |  | 37 | 100% | 29.7 | 28.3 | 33.3 | 0.0, 44.4 |
| Nausea |  | 37 | 100% | 5.0 | 11.7 | 0.0 | 0.0, 0.0 |
| Pain |  | 37 | 100% | 17.6 | 28.0 | 0.0 | 0.0, 33.3 |
| Dyspnoea |  | 37 | 100% | 9.0 | 20.3 | 0.0 | 0.0, 0.0 |
| Insomnia |  | 36 | 97% | 47.2 | 36.8 | 33.3 | 8.3, 66.7 |
| Appetite Loss |  | 37 | 100% | 10.8 | 19.3 | 0.0 | 0.0, 33.3 |
| Constipation |  | 36 | 97% | 8.3 | 16.7 | 0.0 | 0.0, 0.0 |
| Diarrhoea |  | 37 | 100% | 9.0 | 20.3 | 0.0 | 0.0, 0.0 |
| Financial Difficulties |  | 36 | 97% | 18.5 | 33.3 | 0.0 | 0.0, 33.3 |
| **DASS-21** |  |  |  |  |  |  |  |
| Depression |  | 35 | 95% | 4.1 | 4.2 | 3.0 | 1.0, 6.0 |
| Anxiety |  | 36 | 97% | 2.7 | 3.2 | 2.0 | 0.0, 4.0 |
| Stress |  | 37 | 100% | 5.4 | 4.7 | 4.0 | 1.0, 8.0 |
| DASS-21, Depression, Anxiety and Stress 21-item short-form; FCRI-9, Fear of Cancer Recurrence Inventory 9-item short-form; IQR, inter-quartile range; MCQ-12, Melanoma Concerns Questionnaire; QLQ-C30, European Organisation for Research and Treatment of Cancer’s Core Quality of Life Questionnaire; SCNS-34, Supportive Care Needs Survey 34-item short-form; SCNS-M12, Supportive Care Needs Survey Melanoma Module; SCNS-P&C, Supportive Care Needs Survey Partners and Caregivers Module; SD, standard deviation. | | | | | | | |

Supplementary Table 3. Univariable Logistic Regression Results for the Prediction of General Unmet Needs in Melanoma Patients

| Participants Diagnosed with Early-Stage Melanoma | | |
| --- | --- | --- |
| Variable | Odds Ratio (95% CI) | *P* value |
| Age | 0.97 (0.93, 1.01) | **0.126*** |
| Gender (Female) | 1.82 (0.49, 6.74) | 0.371 |
| Family Status (Partnered) | 1.15 (0.30, 4.44) | 0.841 |
| Education |  | 0.790 |
| High school or lower | Reference | |
| University undergraduate degree | 1.46 (0.39, 5.55) | 0.577 |
| University postgraduate degree | 0.92 (0.20, 4.31) | 0.919 |
| Residence (Rural/Remote) | 2.66 (0.52, 13.64) | **0.242*** |
| Income |  | **0.065*** |
| ≤$50,000 AUD | Reference | |
| $50,001-100,000 AUD | 0.12 (0.01, 1.30) | **0.081*** |
| $100,001-200,000 AUD | 0.91 (0.08, 10.21) | 0.935 |
| >$200,000 AUD | 0.23 (0.02, 2.46) | **0.223*** |
| Global Health Status | 0.97 (0.93, 1.01) | **0.088*** |
| Disease Prognosis & Acceptance | 0.77 (0.63, 0.93) | **0.008**** |
| Disease Risk & Future Concerns | 1.23 (1.06, 1.43) | **0.007**** |
| Supportive Care | 0.91 (0.78, 1.07) | **0.229*** |
| Care Delivery & Communication | 0.81 (0.57, 1.15) | **0.241*** |
| Fear of Cancer Recurrence | 1.17 (1.05, 1.31) | **0.004**** |
| Depression | 1.56 (1.04, 2.34) | **0.031**** |
| Anxiety | 2.92 (1.37, 6.23) | **0.005**** |
| Stress | 1.55 (1.19, 2.03) | **0.001**** |
| Participants Diagnosed with Advanced-Stage Melanoma | | |
| Variable | Odds Ratio (95% CI) | *P* value |
| Age | 0.93 (0.89, 0.99) | **0.013**** |
| Gender (Female) | 8.23 (2.20, 30.72) | **0.002**** |
| Family Status (Partnered) | 0.21 (0.03, 1.68) | **0.141*** |
| Education |  | 0.761 |
| High school or lower | Reference | |
| University undergraduate degree | 1.32 (0.37, 4.73) | 0.674 |
| University postgraduate degree | 1.58 (0.45, 5.61) | 0.480 |
| Residence (Rural/Remote) | 3.51 (0.75, 16.42) | **0.111*** |
| Income |  | **0.165*** |
| ≤$50,000 AUD | Reference | |
| $50,001-100,000 AUD | 4.22 (0.73, 24.34) | **0.107*** |
| $100,001-200,000 AUD | 0.80 (0.23, 2.83) | **0.728*** |
| >$200,000 AUD | 2.50 (0.42, 14.83) | 0.313 |
| Global Health Status | **0.93 (0.89, 0.97)** | **<0.001**** |
| Disease Prognosis & Acceptance | **0.72 (0.60, 0.86)** | **<0.001**** |
| Disease Risk & Future Concerns | **1.42 (1.18, 1.71)** | **<0.001**** |
| Supportive Care | **0.81 (0.69, 0.96)** | **0.014**** |
| Care Delivery & Communication | **0.59 (0.40, 0.87)** | **0.008**** |
| Fear of Cancer Recurrence | **1.28 (1.13, 1.45)** | **<0.001**** |
| Depression | **1.90 (1.29, 2.80)** | **0.001**** |
| Anxiety | **2.20 (1.30, 3.70)** | **0.003**** |
| Stress | **1.81 (1.28, 2.55)** | **0.001**** |
| *statistically significant at $\alpha$=0.25.  **statistically significant at $\alpha$=0.05.  CI, confidence interval. | | |

Supplementary Table 4. Univariable Logistic Regression Results for the Prediction of Melanoma-Related Unmet Needs in Melanoma Patients

| Participants Diagnosed with Early-Stage Melanoma | | |
| --- | --- | --- |
| Variable | Odds Ratio (95% CI) | *P* value |
| Age | 0.99 (0.95, 1.02) | 0.430 |
| Gender (Female) | 1.60 (0.46, 5.63) | 0.464 |
| Family Status (Partnered) | 1.06 (0.29, 3.78) | 0.935 |
| Education |  | 0.944 |
| High school or lower | Reference | |
| University undergraduate degree | 1.04 (0.30, 3.58) | 0.954 |
| University postgraduate degree | 0.82 (0.19, 3.58) | 0.788 |
| Residence (rural/remote) | 1.47 (0.39, 5.55) | 0.569 |
| Income |  | 0.733 |
| ≤$50,000 AUD | Reference | |
| $50,001-100,000 AUD | 0.39 (0.06, 2.70) | 0.339 |
| $100,001-200,000 AUD | 0.48 (0.08, 2.95) | 0.429 |
| >$200,000 AUD | 0.75 (0.10, 5.47) | 0.777 |
| Global Health Status | 0.96 (0.93, 1.00) | **0.049**** |
| Disease Prognosis & Acceptance | 0.79 (0.66, 0.94) | **0.007**** |
| Disease Risk & Future Concerns | 1.18 (1.04, 1.34) | **0.013**** |
| Supportive Care | 0.91 (0.79, 1.05) | **0.187*** |
| Care Delivery & Communication | 0.68 (0.48, 0.97) | **0.029**** |
| Fear of Cancer Recurrence | 1.09 (1.00, 1.18) | **0.056*** |
| Depression | 1.04 (0.87, 1.24) | 0.650 |
| Anxiety | 1.83 (1.20, 2.80) | **0.005**** |
| Stress | 1.24 (1.04, 1.48) | **0.018**** |
| Participants Diagnosed with Advanced-Stage Melanoma | | |
| Variable | Odds Ratio (95% CI) | *P* value |
| Age | 0.98 (0.95, 1.02) | 0.333 |
| Gender (Female) | **2.86 (1.23, 6.61)** | **0.014**** |
| Family Status (Partnered) | 0.99 (0.35, 2.79) | 0.987 |
| Education |  | 0.902 |
| High school or lower | Reference | |
| University undergraduate degree | 1.20 (0.43, 3.36) | 0.729 |
| University postgraduate degree | 0.93 (0.36, 2.42) | 0.877 |
| Residence (rural/remote) | 1.24 (0.49, 3.15) | 0.644 |
| Income |  | 0.794 |
| ≤$50,000 AUD | Reference | |
| $50,001-100,000 AUD | 1.66 (0.52, 5.36) | 0.392 |
| $100,001-200,000 AUD | 1.10 (0.36, 3.35) | 0.872 |
| >$200,000 AUD | 1.50 (0.41, 5.54) | 0.543 |
| Global Health Status | **0.94 (0.91, 0.96)** | **<0.001**** |
| Disease Prognosis & Acceptance | **0.78 (0.68, 0.89)** | **<0.001**** |
| Disease Risk & Future Concerns | **1.27 (1.23, 1.43)** | **<0.001**** |
| Supportive Care | **0.76 (0.66, 0.87)** | **<0.001**** |
| Care Delivery & Communication | **0.58 (0.44, 0.77)** | **<0.001**** |
| Fear of Cancer Recurrence | **1.13 (1.05, 1.21)** | **0.001*** |
| Depression | **1.51 (1.23, 1.84)** | **<0.001**** |
| Anxiety | **1.61 (1.24, 2.08)** | **<0.001**** |
| Stress | **1.35 (1.16, 1.56)** | **<0.001**** |
| *statistically significant at $\alpha$=0.25.  **statistically significant at $\alpha$=0.05.  CI, confidence interval. | | |
